# The ROX@PDA@PCL vascularized bionic nerve conduit facilitates the restoration of nerve defects

**DOI:** 10.3389/fneur.2025.1561177

**Published:** 2025-05-13

**Authors:** Daoyi Lin, Jun Peng, Yichong Zhang, Xiaoping Wang, Xiaodong Xu, Jing Jia

**Affiliations:** ^1^Department of Trauma and Orthopedic, China-Japan Friendship Hospital, Beijing, China; ^2^Department of Anesthesia, China-Japan Friendship Hospital, Beijing, China; ^3^Department of Orthopedic, The Ninth Medical Center of Chinese PLA General Hospital, Beijing, China; ^4^Department of Trauma and Orthopedic, Peking University People’s Hospital, Beijing, China; ^5^Department of Outpatient, The Ninth Medical Center of Chinese PLA General Hospital, Beijing, China

**Keywords:** roxadustat, nerve defect, nerve conduit, vascularization, neurological function

## Abstract

Previous research has highlighted the pivotal role of angiogenesis in facilitating nerve function repair following nerve injury. In this study, we employed polydopamine (PDA) to modify polycaprolactone (PCL) and subsequently loaded it with roxadustat (ROX), thereby constructing a vascularized nerve conduit for the repair of a 10 mm sciatic nerve defect in rats. At 2 weeks post-surgery, new blood vessels were evaluated by immunofluorescence staining. Twelve weeks post-surgery, a comprehensive suite of assessments was conducted to evaluate the efficacy of the conduit, including gait analysis, determination of gastrocnemius muscle wet weight recovery, electrophysiological examination of gastrocnemius compound action potential (CMAP), Masson staining to evaluate gastrocnemius muscle fiber cross-sectional area, toluidine blue staining to assess the total number of regenerated myelinated nerve fibers, and electron microscopic observation of myelin sheath thickness. Our findings revealed that ROX@PDA@PCL could promote the proliferation of vascular endothelial cells and significantly enhance angiogenesis in regenerated nerves (*p* < 0.05). Regarding the recovery of neurological function, compared to the PDA@PCL and PCL groups, the ROX@PDA@PCL group exhibited significantly superior outcomes in the sciatic functional index (SFI), CMAP, gastrocnemius muscle wet weight ratio, muscle fiber cross-sectional area, total number of regenerated myelinated nerve fibers, and myelin sheath thickness. These indices approached those of the autologous group, but were still lower than in the autograft group (*p* < 0.05). The study underscores the potential of the vascularized nerve graft (ROX@PDA@PCL), constructed through PDA-mediated loading of ROX onto PCL, to enhance functional nerve recovery. Our findings present a promising new therapeutic approach for the clinical repair of peripheral nerve defects.

## Introduction

Large peripheral nerve defects constitute a prevalent clinical challenge (1). When the peripheral nerve sustains damage, the innervated area experiences sensory impairment, motor dysfunction, and muscular atrophy, among other sequelae (2). Inadequate management of such injuries can lead to lifelong disability, a high disability rate, and a considerable familial and societal burden. In response, scholars (3, 4) have sequentially explored the use of artificial tissue-engineered nerves, acellular allogeneic nerves, and artificial nerves enriched with diverse bioactive substances to mend extensive segmental defects of peripheral nerves, achieving notable repair outcomes.

Among these innovations, the polycaprolactone (PCL) conduit stands out as a nerve graft that has garnered clinical validation (5, 6). However, a significant limitation persists: tissue-engineered nerves lack an independent vascular supply. Their blood nourishment primarily relies on the infiltration of adjacent tissue fluid to replenish the internal nutrition of the nerve graft. Yet, mere infiltration falls short of meeting the extensive nutritional demands for axonal regeneration and growth, significantly impeding the restoration of nerve function.

Recent research has illuminated that the aggregation of vascular endothelial cells serves as the ‘initiator’ in the remodeling of scaffolds (7). The blood vessels within the microenvironment play a pivotal role in the nerve regeneration process (8, 9). They function not merely as conduits for nutrient diffusion, cell proliferation, and axon growth but also exert a crucial regulatory influence on cells and signaling molecules involved in axon regeneration. Consequently, there is an urgent imperative to address the blood supply issue in nerve grafts. To enhance the vascular regeneration capabilities of tissue-engineered nerves, addressing the intricacies of the vascular regeneration microenvironment is paramount. Vascular endothelial cells stand as the seed cells, possessing the potential to catalyze the rejuvenation of nerve grafts. However, in the event of extensive or long-distance peripheral nerve defects, the quantity of these vital cells within the injured area becomes woefully inadequate. Various stem cells hold the promise of differentiating into vascular endothelial cells (10, 11), thereby serving as prime candidates for tissue-engineered nerve applications. Yet, the debate persists regarding the ultimate efficacy and biosafety of stem cells within a living organism. Consequently, the pursuit of a secure method to augment the proliferation of vascular endothelial cells represents a pivotal research orientation in the field of bionic nerves. Therefore, enhancing the ability of tissue-engineered nerve conduits to promote vascular regeneration may be a critical approach to improving the recovery of neurological functions.

Roxadustat (ROX), a small molecule compound that has garnered approval from both the United States and Europe, has emerged as a safe and effective treatment for the clinical management of renal anemia (12, 13). Prior investigations have illuminated its capacity to foster angiogenesis, primarily through the induction of HIF-*α* expression (14). However, the role of ROX in promoting neurovascular regeneration remains unclear.

Therefore, this study combines the clinically widely-used pro-angiogenic ROX with PCL conduits, which possess nerve-bridging capabilities through polydopamine (PDA) modification to construct a vascularized neural scaffold. This approach aims to enhance the vascular regeneration-promoting capacity of PCL conduits, thereby improving the repair efficacy of nerve defects through promoted vascular regeneration, ultimately providing a novel therapeutic strategy for clinical nerve.

## Materials and methods

### Animals

Forty-eight specific-pathogen-free (SPF), six-week-old female Sprague Dawley rats were maintained in a controlled environment, featuring a 12-h light/dark cycle and a humidity level of 40%. All experimental procedures were rigorously adhered to the Animal Welfare Code and received approval from the Animal Ethics Committee of China-Japan Friendship Hospital (Approval Number: ZRDWLL240049).

### Preparation of ROX@PDA@PCL nerve conduit

Firstly, PCL nerve conduit was prepared by electrospinning, and the specific preparation method was referred to Frost HK ‘s literature (15). Secondly, a 10 mmol/L Tris–HCl buffer solution (pH 8.5) was prepared. Dopamine hydrochloride (Merk, Germany) was then added to achieve a concentration of 2 mg/mL. The PCL conduit was submerged in this solution and subjected to a 24-h reaction in a 37°C constant-temperature oscillator at 60 revolutions per minute, resulting in the formation of PCL@PDA. Lastly, roxadustat (ROX, Selleck, China) was dissolved in Tris–HCl buffer (1 mg/mL), and PCL@PDA was immersed in this solution for an additional 24-h reaction period to produce ROX@PDA@PCL.

### ROX release curve

Three nerve conduits of ROX@PDA@PCL were immersed in 5 mL of PBS at 37°C as the experimental group, while the same number of PDA@PCL nerve conduits served as the blank control. Each group contained five parallel samples. During the two-week incubation period, 3 mL of PBS containing released ROX was collected daily, and an equal volume of fresh PBS was replenished after each sampling. The absorbance of ROX solutions at different concentrations was measured at 274 nm using a UV–Vis spectrophotometer. A standard curve was constructed with ROX concentration on the *x*-axis and absorbance on the *y*-axis. The absorbance of the collected samples was measured at 274 nm, and the net absorbance of released ROX was calculated as (OD of the experimental group – OD of the control group). The ROX concentration in each sample was determined using the standard curve. The cumulative release rate of ROX at different time points was calculated as [(cumulative ROX release at the corresponding time point − PDA@PCL)/total ROX in the material].

### CCK-8 test

Five nerve conduits of PCL, PDA@PCL, and ROX@PDA@PCL were taken, respectively, and placed into 15 mL centrifuge tubes. Eight milliliter of culture medium containing 2% FBS was added to the centrifuge tubes, and after extraction for 72 h, the supernatant was taken for culturing human umbilical vein endothelial (HUVEC) cells. Well-growing HUVEC cells were inoculated in 96-well plates with a cell seeding density of approximately 5,000 cells per well. After the cells adhered for 6–8 h, the medium after extracting PCL, PDA@PCL, ROX@PDA@PCL and normal medium as Blank control, were added to each group with 5 replicate wells, and the experiment was repeated 3 times. At 48 h intervention, 10 μL of CCK-8 (Dongren Chemical Technology Co., Ltd. Japan) was added to each well, and the cells were incubated at 37°C for 2 h. The supernatant was aspirated and added to a new 96-well plate. The absorbance value of each well (with a wavelength of 450 nm) was detected by a microplate reader (Bio-Rad, United States), and the absorbance (OD value) was recorded.

### Animal model

**Figure 1:**
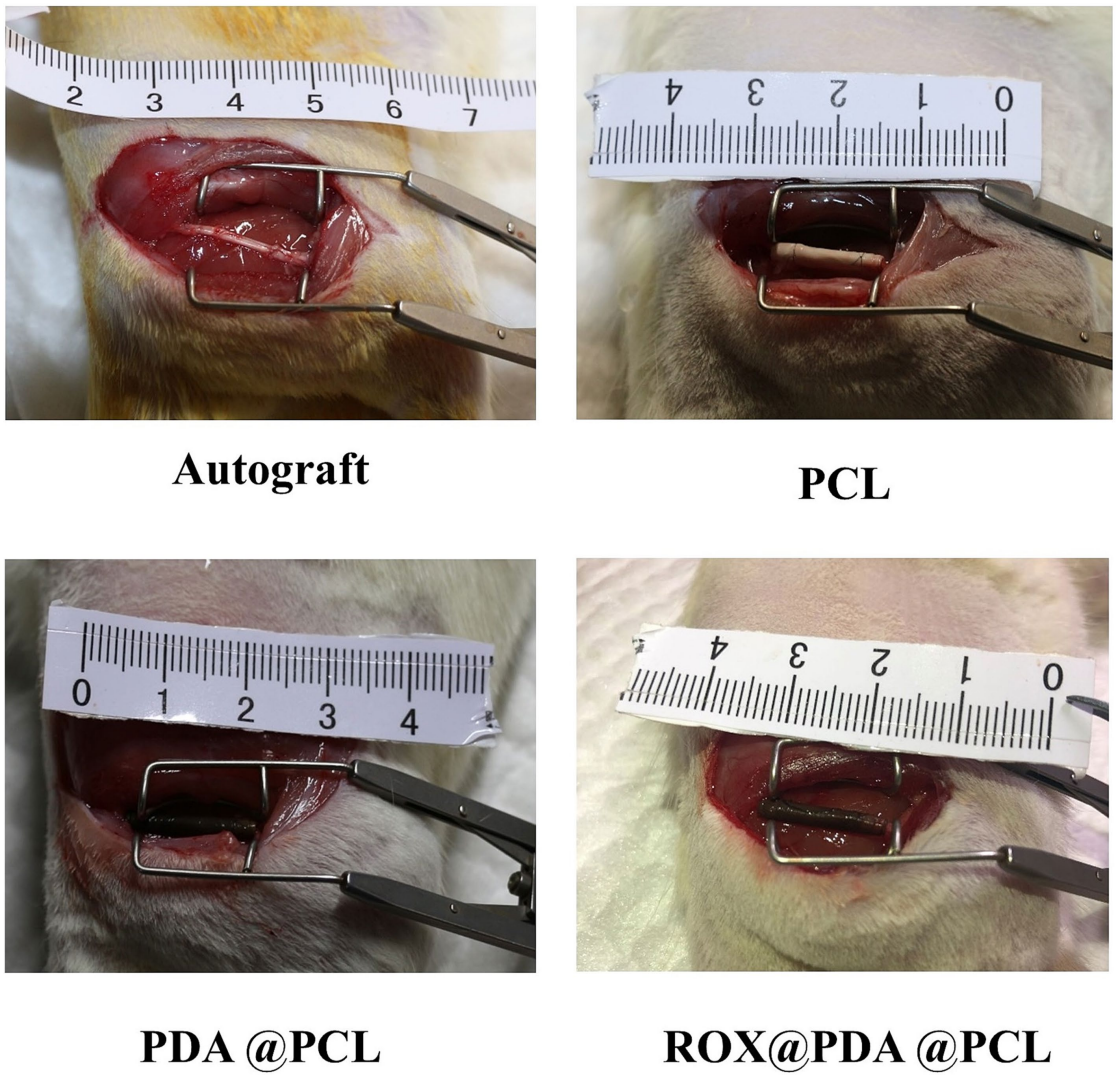
Surgical overseen (*n* = 12). The PCL conduit is white, and the dopamine-modified PCL conduit becomes black.

The rats were randomly divided into 4 groups (*n* = 12) in each group (Autograft, PCL, PDA@PCL and ROX@PDA@PCL group). The operation process was as follows: anesthesia was induced by 5% isoflurane, skin was prepared on the right thigh, and the right sciatic nerve was exposed after disinfection. Sciatic nerve transection injury was caused by cutting the sciatic nerve from the lower edge of the piriformis. Under the microscope, the nerve conduit was micro-sutured into the sciatic nerve stump of rats with a 10–0 nylon suture, with a break spacing of 10 mm. Autograft group was *in situ* anastomosis by epineurium suture. Finally, the surgical site was rinsed with normal saline and the muscles and skin were sutured layer by layer (Figure 1).

### Immunofluorescence staining

Two weeks after operation, the regenerated sciatic nerves were collected (*n* = 6). Samples were fixed in 4% paraformaldehyde for 12 h. The sample was dehydrated in 20 and 30% sucrose for 12 h, and then sliced to a 10 μm-thick piece by a frozen slicer (Leica CM 1950, German). The 10um sections were slide mounted for staining. The sections were rinsed in PBS and incubated in goat serum working solution for 30 min. Then, samples were incubated at 4°C overnight in CD31 and *α*-SMA (α-smooth muscle actin) antibody working solution. The next day, samples were rinsed using PBS and incubated in a secondary antibody working solution for 2 h at 24°C. Further, samples were rinsed thrice using PBS, after which the nuclei were stained with DAPI, sealed with an aqueous sealer, and imaged using a laser confocal microscope. To evaluate blood vessel regeneration, three visual fields at 20× magnification were randomly selected for each sample. The areas of CD31^+^ and *α*-SMA^+^ positive regions were quantified using Image J Pro Plus software (NIH, United States), expressed as a percentage of the total selected region area. A larger positive area percentage is generally interpreted as indicating greater neovascularization density and higher vessel maturity. The specific antibodies used and their working concentrations are listed in Table 1.

**Table 1:** The working concentration of antibody.

Antibody	Manufacturers	Product code	Concentration
Mouse anti-α-SMA antibody	Abcam	ab7817	1:200
Rabbit anti-CD31 antibody	Abcam	ab222783	1:100
Goat Anti-Rabbit IgG H&L (Alexa Fluor^®^ 594)	Abcam	ab150080	1:200
Goat Anti-Mouse IgG H&L (Alexa Fluor^®^ 488)	Abcam	ab150117	1:200
4,6-diamidino-2-henylindole (DAPI)	4A Biotech	39–100	1:50

### Sciatic functional index (SFI)

The SFI was assessed in the experimental rats at 12 weeks post-operation. For gait analysis, the CatWalk XT 10.5 system (Noldus, Netherlands) was initiated, with the treadmill width adjusted according to the rats’ size to ensure accuracy. Following defecation and urination, the rats from each group were positioned on the left side of the CatWalk XT gait analyzer, allowing them to spontaneously run to the right end. The system automatically captured and recorded pertinent motion parameters, subsequently calculating the SFI for the right hind limb. The calculation formula of SFI is 109.5 (ETS–NTS)/NTS – 38.3 (EPL–NPL)/NPL + 13.3 (EIT–NIT)/NIT – 8.8. N: normal; E: experimental; TS: the transverse distance between the first and the fifth toes; PL: print length from heel to longest toe; IT: the transverse distance between the second and fourth toes.

### Nerve electrophysiology

After the gait analysis, the rats were anesthetized using isoflurane gas at a concentration of 2.5% and a flow rate of 100 mL/min. The skin was incised along the previous surgical scar, facilitating the blunt dissection of the sciatic nerve and the exposure of the anterior tibial and gastrocnemius muscles. Stimulation electrodes were positioned approximately 5 mm apart at the distal and proximal ends of the nerve stump, while the induction electrode was placed on the gastrocnemius muscle. The Synergy electrophysiological instrument (Oxford, United Kingdom) was configured to deliver rectangular pulses with a duration of 0.1 ms, a current intensity of 0.09 mA, and a frequency of 1 Hz. Each pulse was meticulously recorded, and the differences in time and latency between adjacent distal and proximal conduction times were documented and computed. Subsequently, the difference in sciatic nerve conduction velocity was determined.

### Toluidine blue staining of regenerated nerve fibers

At 12 weeks postoperatively, the distal segments of regenerated nerve grafts were fixed in 4% paraformaldehyde for 24 h, then 1 μm thick cross-sections were cut using an ultrathin slicer (Diatome Ultra45^。^, Switzerland). After rinsing with tap water, the sections were immersed in 0.1% toluidine blue staining solution for 5 min, followed by rinsing with water to remove excess dye. Dehydration was performed using alcohol, followed by clearing with xylene, and then slides were sealed. Photographs were taken using a microscope (Nikon, Japan) under 10× object glass. Image Pro Plus software was used to count the total number of regenerated myelinated nerve fibers.

### Transmission electron microscopy of regenerated nerve fibers

At 12 weeks postoperatively, the distal segments of regenerated nerve grafts were fixed in 2.5% glutaraldehyde for 24 h. Ultra-thin sections with a thickness of 70 nm were obtained using an ultrathin slicer. Staining was performed using a 3% concentration of lead citrate and uranyl acetate in copper grids with polyvinyl alcohol-coated/carbon support membranes. Transmission electron microscopy (Hitachi HT7800, Japan) was used to observe the myelin sheath under a voltage of 80 kV and magnification of 1,000–5000×. One hundred myelinated nerve fibers were randomly selected for each sample, and Image J Pro Plus software (NIH, United States) was used to measure the thickness of the myelin sheath and the diameter of the myelinated fibers for statistical analysis.

### Muscle weight

After the rats were sacrificed, the gastrocnemius muscles from both hind limbs were harvested. The muscle surface was cleaned with gauze, and the weight was immediately measured using an electronic balance. The ratio of the wet weight of the affected limb’s gastrocnemius muscle to that of the healthy limb was then calculated.

### Muscle MASSON staining

After measuring the wet weight of the muscle, the muscle was placed in 4% paraformaldehyde for fixation 24 h, and then dehydrated successively with 50, 70, 90, and 100% ethanol for 30 min per step. The muscle was embedded in paraffin, and sliced into a 7 μm-thick piece. The 7 μm sections were slide mounted for Masson staining (Solarbio, China). The nuclei were stained with 0.2% hematoxylin for 5 min, washed, and then stained with Masson’s red acid fuchsin for 5 min. The sections were briefly washed with a 2% glacial acetic acid aqueous solution. Differentiation was performed using 1% phosphomolybdic acid aqueous solution for 3–5 min. The sections were then stained directly with 2% aniline blue or 1% light green solution for 5 min without washing. Afterward, the sections were briefly washed with a 0.2% glacial acetic acid aqueous solution. Dehydration was performed using 95% alcohol, followed by anhydrous alcohol, and the sections were cleared with xylene. Finally, the slides were sealed with neutral gum. After sealing, the slides were imaged under a microscope (Nikon, Japan). The cross-sectional area of muscle fibers in the abdominal portion of the gastrocnemius muscle was calculated using ImageJ software. Three visual fields at 20× magnification were selected for each sample, and the average value was used as the ultimate muscle fiber cross-sectional area for the sample. The calculation formula of mean muscle fiber cross-sectional area is that the selected visual field area is divided by the number of muscle fiber fibers.

### Statistical analysis

Data analysis was conducted using GraphPad Prism V.9.0 (San Diego, CA, United States). All quantitative data were obtained from at least three independent experiments and are expressed as the mean ± standard deviation (SD). Single comparisons were performed using unpaired t-tests, while multiple comparisons were conducted using one-way analysis of variance (ANOVA) followed by Tukey’s *post hoc* test. The minimum significance level was set at *p* < 0.05 (two-tailed).

## Results

### Characterization of ROX@PDA@PCL nerve conduit

**Figure 2:**
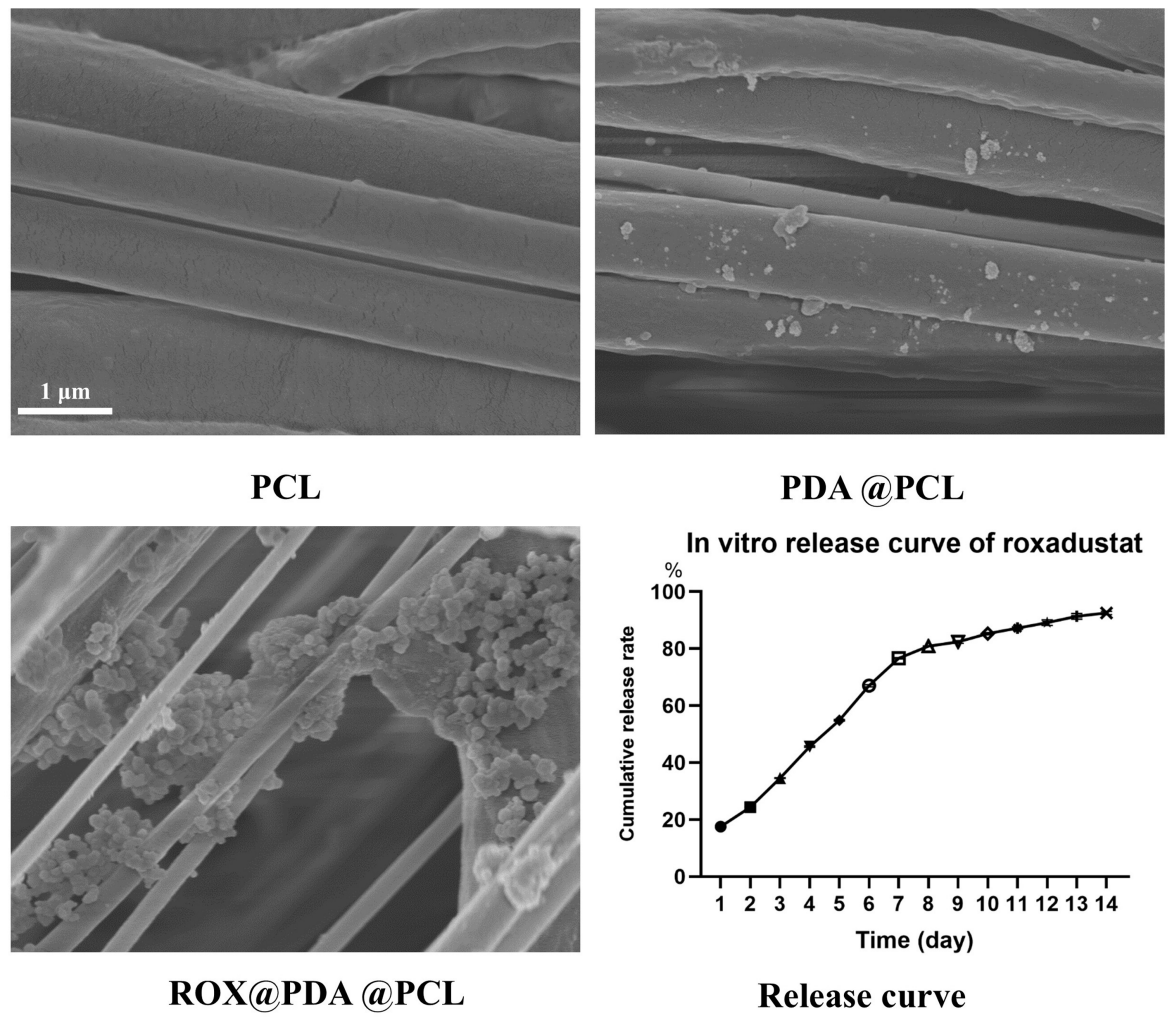
The morphological characterization of nerve conduits via scanning electron microscopy (SEM) and the sustained-release profile of ROX are presented. The SEM images (*n* = 3) demonstrate that polycaprolactone (PCL) nanofibers constituting the nerve conduits exhibit parallel alignment along a unified orientation. Polydopamine (PDA)-modified PCL nerve conduits display adherent PDA particles on their surfaces, whereas ROX-loaded PDA@PCL nerve conduits exhibit substantial granular deposits corresponding to ROX bound to PDA matrices. In the *in vitro* ROX release profile (*n* = 6), the cumulative release percentage (*Y*-axis) progressively increases with temporal progression (*X*-axis). The cumulative release percentage was calculated as the ratio of total drug released at specific time intervals to the total drug loading amount in the nerve conduits. Distinct symbols on the release curve denote percentage values of cumulative drug release at designated time points.

The wall thickness of the PCL, PDA@PCL, and ROX@PDA@PCL nerve conduits was approximately 200 μm. Both PDA and ROX@PDA had minimal effects on the wall thickness. Scanning electron microscopy (SEM) images revealed that PCL fiber bundles exhibited clear orientation, with PDA and ROX visible on the surface of the PCL fiber bundles (Figure 2).

The sustained-release curve demonstrated that ROX@PDA@PCL (*n* = 6) allowed for continuous, slow release of ROX. A rapid release of 76.66 ± 0.25% occurred within the first 7 days, followed by a gradual decline in the release rate. The cumulative release reached 92.53 ± 0.62% within 14 days (Figure 2).

### Cell proliferation assay

**Figure 3:**
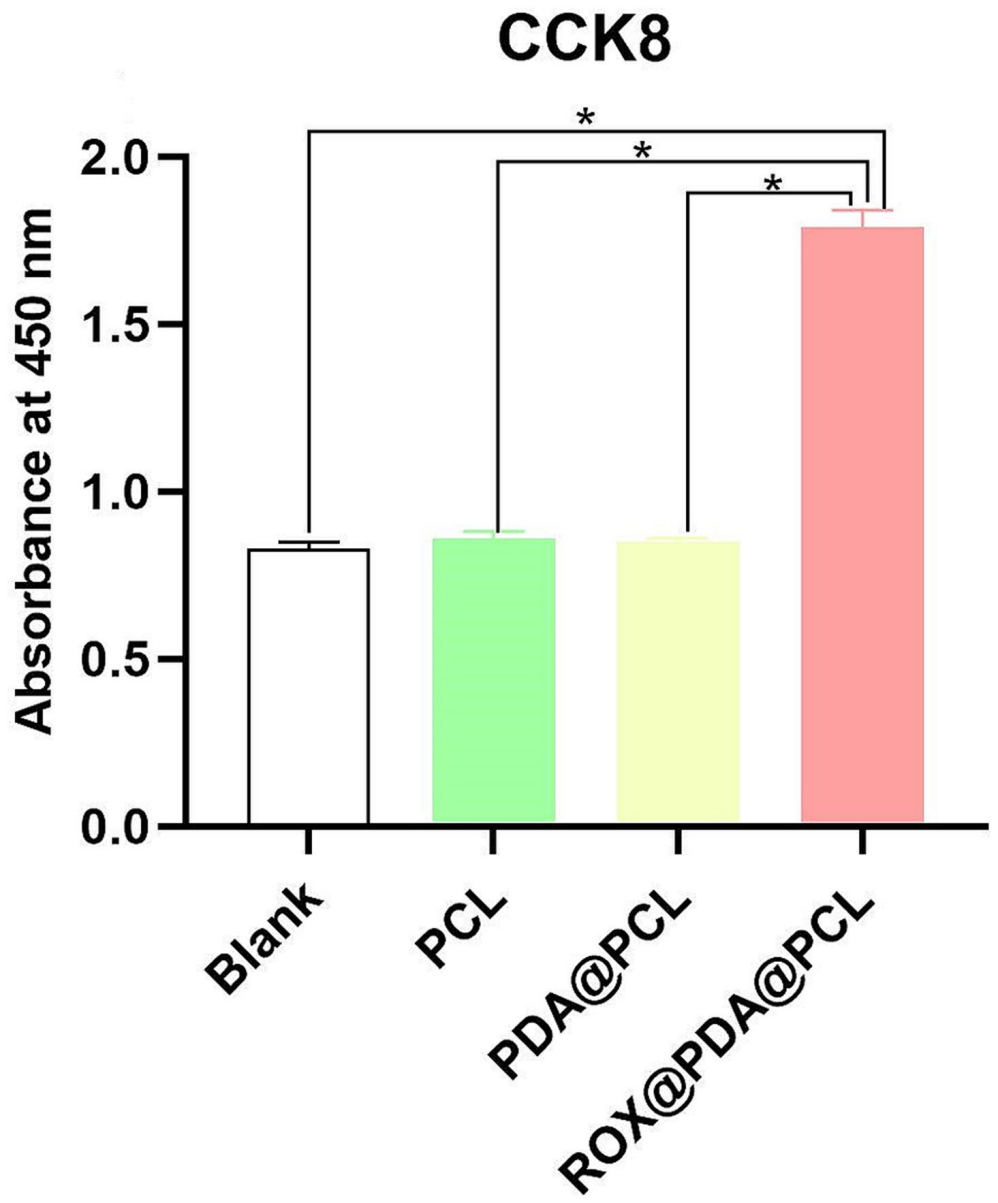
Absorbance values of HUVEC of CCK8 test at 48 h after co-culture. Absorbance value (*Y*-axis) is an arbitrary unit. Data are expressed as mean ± SD, *n* = 3. *p < 0.05.

After HUVEC cells were cultured for 48 h, the absorbance values for the Blank (*n* = 3), Autograft (*n* = 3), PCL (*n* = 3), and PDA@PCL (*n* = 3) groups were 0.83 ± 0.02, 0.84 ± 0.01, 0.84 ± 0.02, and 0.85 ± 0.01, respectively, with no significant differences between them. The absorbance for the ROX@PDA@PCL group (*n* = 3) was 1.79 ± 0.05, which was significantly higher than that of the other four groups (*p* < 0.005) (Figure 3).

### Vascular regeneration

**Figure 4:**
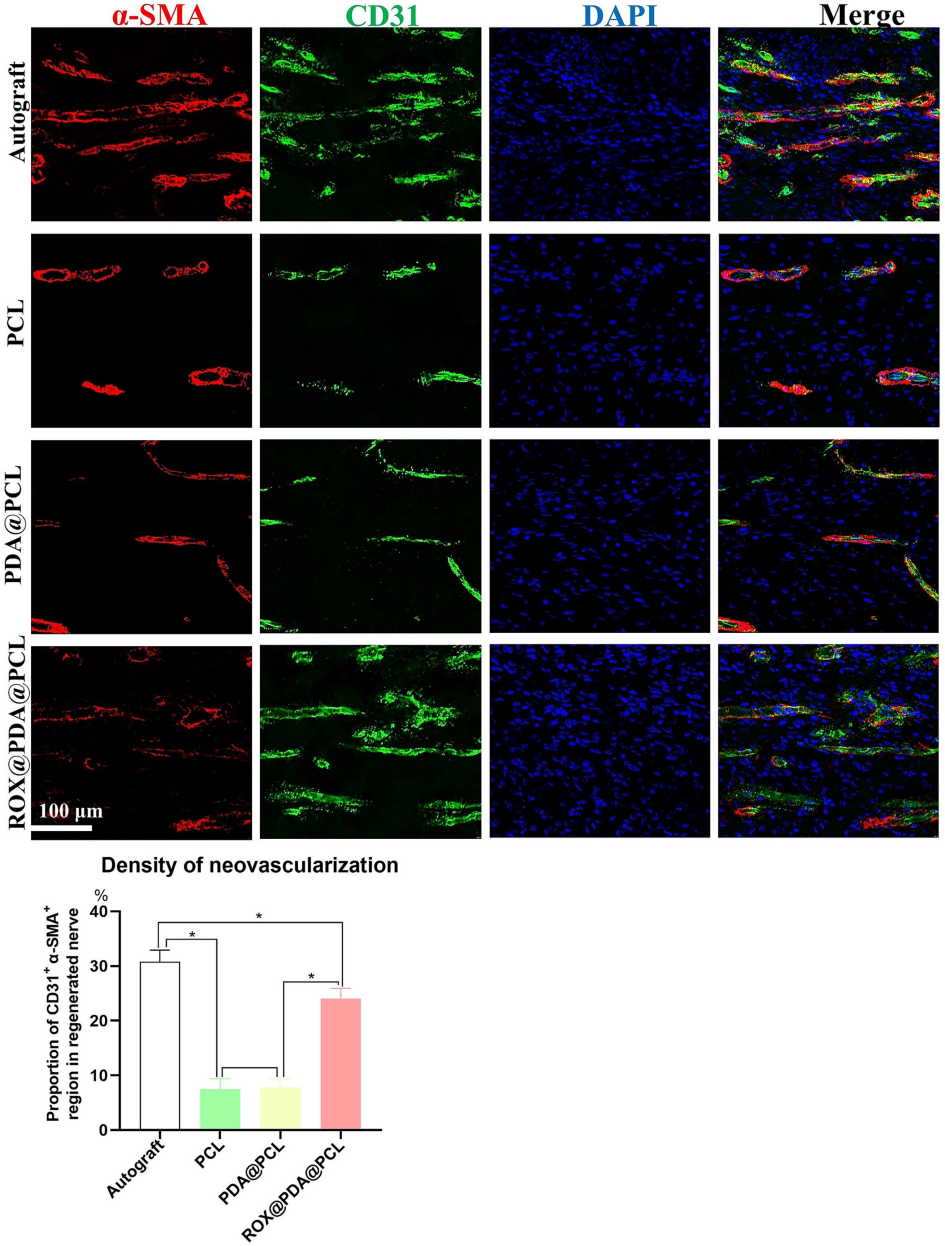
Regeneration of blood vessels of regenerated nerves. The density of regenerative vessels in group ROX@PDA@PCL was significantly higher than that of the groups PCL and PDA@PCL. Red is *α*-SMA, green is CD31, Blue is DAPI. Data are expressed as mean ± SD, *n* = 6. *p < 0.05.

At 2 weeks post-operation, varying densities of neovascularization were observed in the regenerated nerves of the four groups. The proportion of *α*-SMA^+^ CD31^+^ regions in the Autograft (*n* = 6), PCL (*n* = 6), PDA@PCL (*n* = 6), and ROX@PDA@PCL (*n* = 6) groups were 30.8 ± 2.1%, 7.5 ± 1.9%, 7.8 ± 1.5%, and 24.0 ± 1.9%, respectively. The proportion of α-SMA^+^ CD31^+^ in the ROX@PDA@PCL group was significantly higher than in the other conduit groups (*p* < 0.05), but significantly lower than in the Autograft group (*p* < 0.05) (Figure 4).

### Recovery of hind limb function

**Figure 5:**
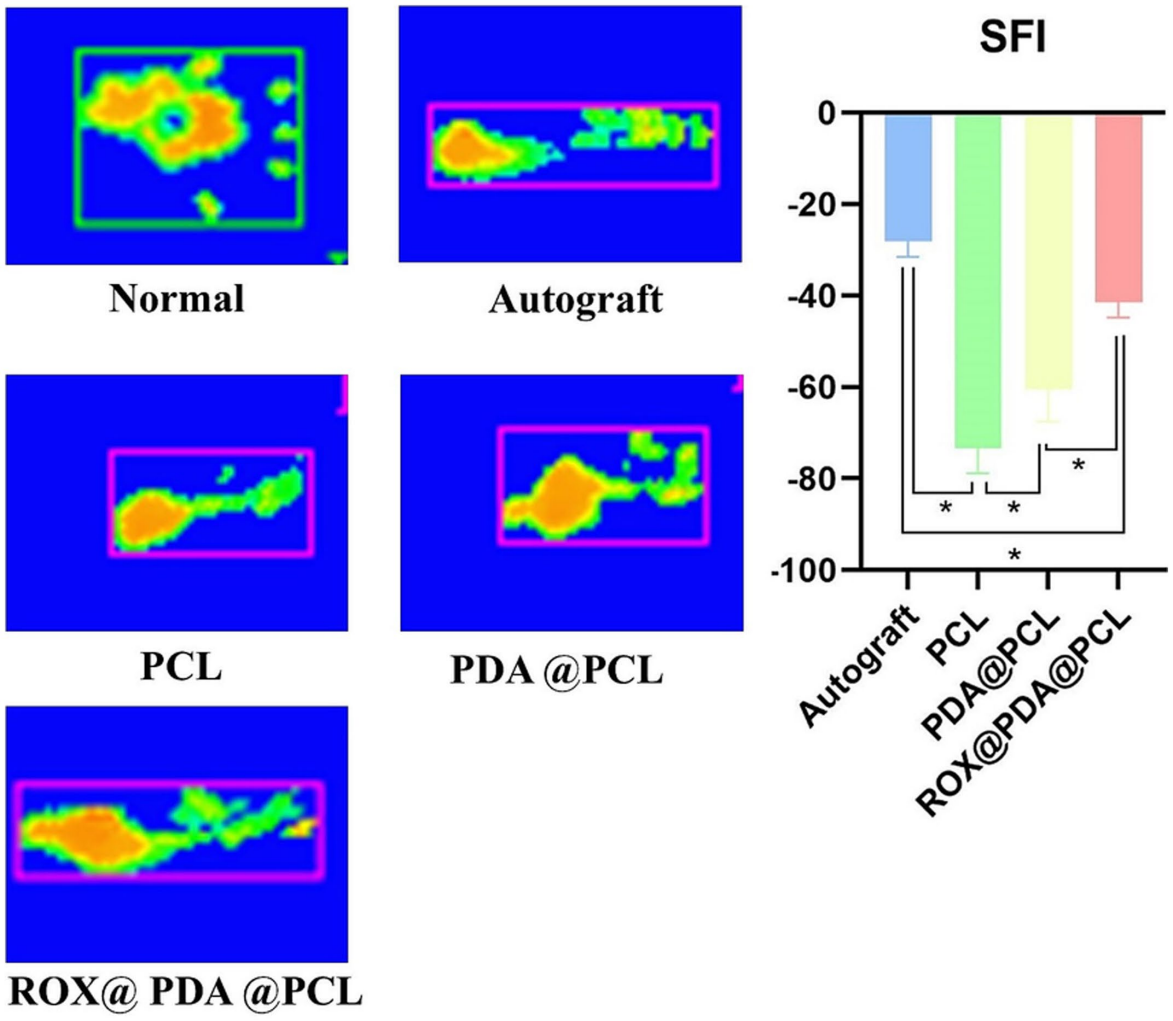
Rat’s affected limb sciatic functional index (SFI). The Gait recording system (Catwalk) can accurately record the footprint morphology of rats and calculate the SFI. Scores approaching 0 reflect optimal restoration of neurological function, as quantified by the SFI. The green parts of the footprints indicate areas with lower pressure, while the yellow parts indicate areas with higher pressure. Data are expressed as mean ± SD, *n* = 6. *p < 0.05.

At 12 weeks post-operation, the right hind limbs of rats in each group (*n* = 6) exhibited varying degrees of atrophy. The sciatic nerve function index for the Autograft group (−28.25 ± 3.27) was significantly better than that of the other three groups (*p* < 0.05). The sciatic nerve function indexes for the ROX@PDA@PCL, PDA@PCL, and PCL groups were −41.5 ± 3.33, −60.45 ± 7.11, and −73.45 ± 5.33, respectively, with significant statistical differences between these groups (*p* < 0.05). The plantar pressure distribution during walking revealed that normal rats exhibited even plantar stress across the entire paw. The Autograft and ROX@PDA@PCL groups showed a two-point force distribution, while only heel force was observed in the PDA@PCL and PCL groups (Figure 5).

**Figure 6:**
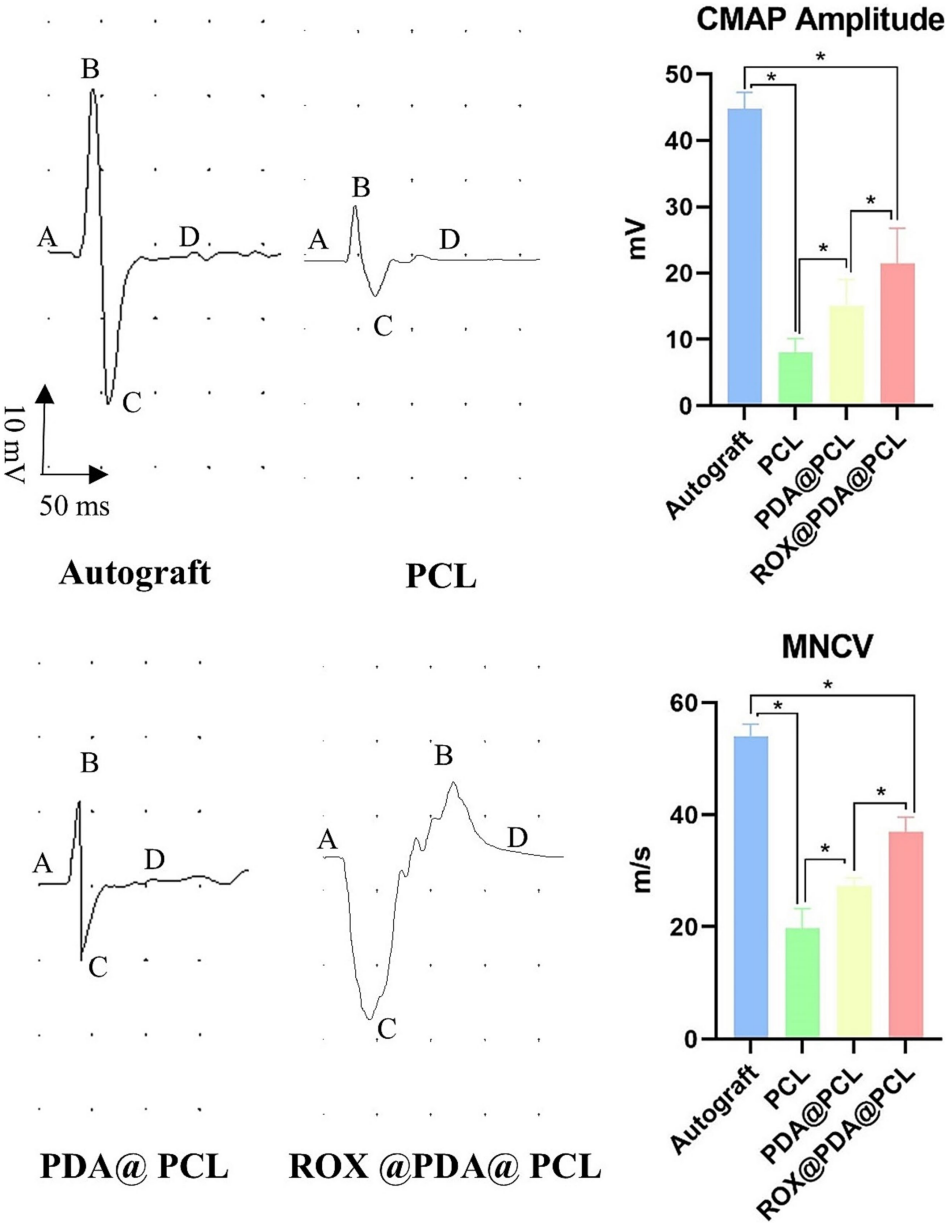
Nerve conduction function. Normal “sinusoidal” waveform could be restored in all groups after nerve injury repair, and the amplitude of CMAP and nerve conduction velocity in group ROX@PDA@PCL were significantly higher than that in group PDA@PCL and PCL. The segment between points A and D corresponds to the compound muscle action potential (CMAP), where B represents the peak amplitude, and C denotes the trough. The magnitude of the CMAP is defined as the voltage difference between points B and C. Data are expressed as mean ± SD, *n* = 6. *p < 0.05.

At 12 weeks post-surgery, the nerve conduction velocities for the Autograft (*n* = 6), ROX@PDA@PCL (*n* = 6), PDA@PCL (*n* = 6), and PCL (*n* = 6) groups were 54 ± 2.16 m/s, 37 ± 2.58 m/s, 27 ± 1.5 m/s, and 20 ± 3.5 m/s, respectively. The CMAP amplitudes for the same groups were 44.75 ± 2.5 mV, 21.50 ± 5.26 mV, 15.00 ± 4.08 mV, and 8.00 ± 2.16 mV, respectively. The nerve conduction velocity and CMAP amplitudes for the ROX@PDA@PCL group were significantly higher than those for the PDA@PCL and PCL groups (*p* < 0.05), but slightly lower than those for the Autograft group (*p* < 0.05) (Figure 6).

### Nerve tissue recovery

**Figure 7:**
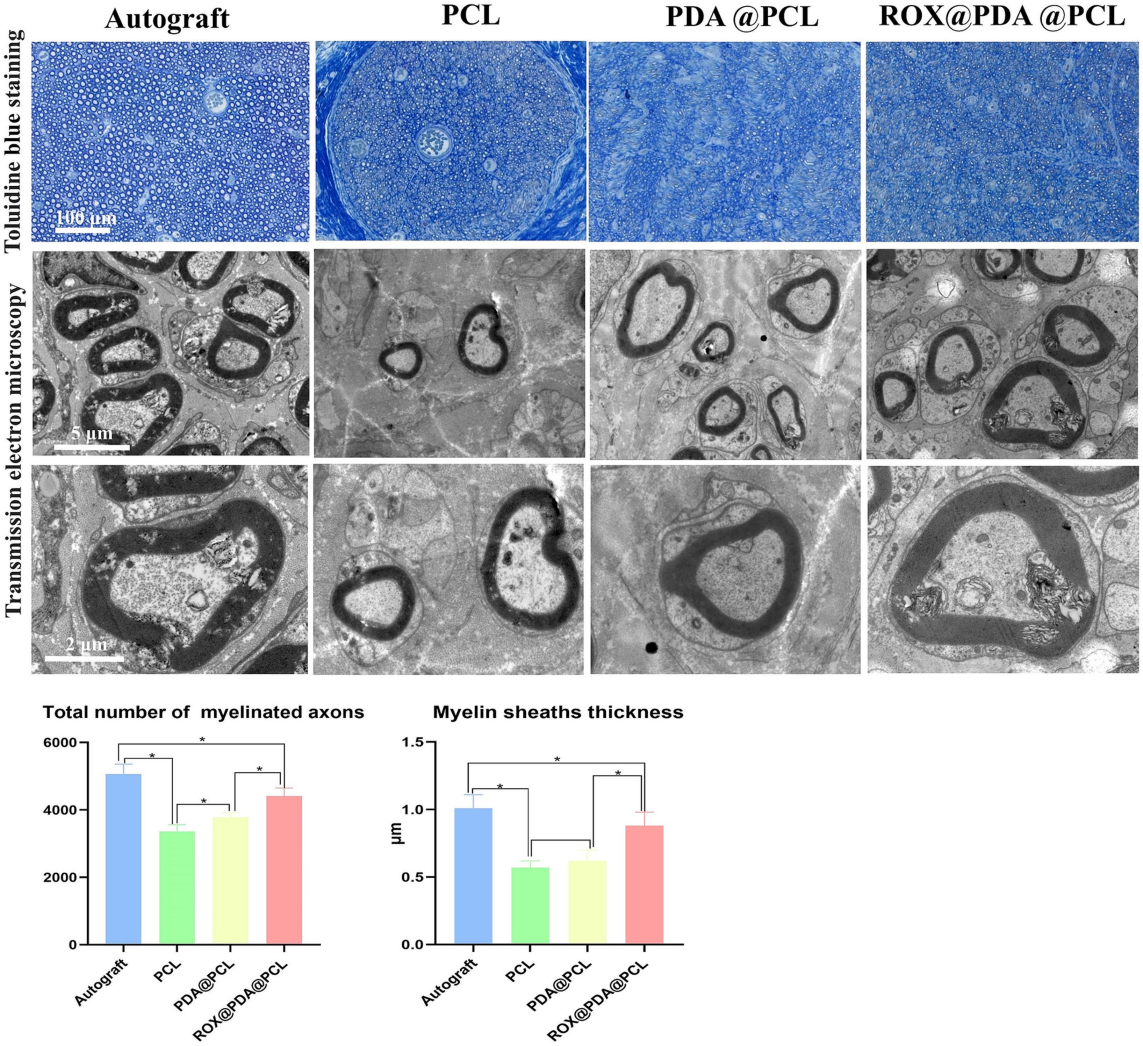
Toluidine blue staining and TEM scanning of regenerated myelinated nerve fibers. Data are expressed as mean ± SD, *n* = 6. *p < 0.05.

At 12 weeks after surgery, toluidine blue staining revealed that the number of myelinated nerve fibers in the Autograft (*n* = 6), ROX@PDA@PCL (*n* = 6), PDA@PCL (*n* = 6), and PCL (*n* = 6) groups were 5,069 ± 288, 4,416 ± 237, 3,788 ± 131, and 3,358 ± 202, respectively. The ROX@PDA@PCL group had significantly more myelinated fibers than the other conduit groups but still fewer than the Autograft group (*p* < 0.05). Transmission electron microscopy showed the following myelin sheath thicknesses for the regenerated nerve fibers in the Autograft (*n* = 6), ROX@PDA@PCL (*n* = 6), PDA@PCL (*n* = 6), and PCL (*n* = 6) groups were 1.01 ± 0.10 μm, 0.88 ± 0.10 μm, 0.62 ± 0.08 μm, and 0.57 ± 0.05 μm. The myelin sheath thickness for the ROX@PDA@PCL group was significantly higher than the other conduit groups (*p* < 0.05), but significantly lower than the Autograft group (*p* < 0.05). No significant difference was observed between the PCL and PDA@PCL groups (*p* > 0.05) (Figure 7).

### Muscle tissue recovery

At 12 weeks after surgery, the muscle wet weight ratios for the Autograft (*n* = 6), ROX@PDA@PCL (*n* = 6), PDA@PCL (*n* = 6), and PCL (*n* = 6) groups were 77.21 ± 4.23%, 59.47 ± 4.01%, 42.58 ± 2.99%, and 37.67 ± 2.23%, respectively. The ROX@PDA@PCL group had a significantly higher muscle wet weight ratio than the other conduit groups (*p* < 0.05), with no significant difference between the PCL and PDA@PCL groups (*p* > 0.05).

**Figure 8:**
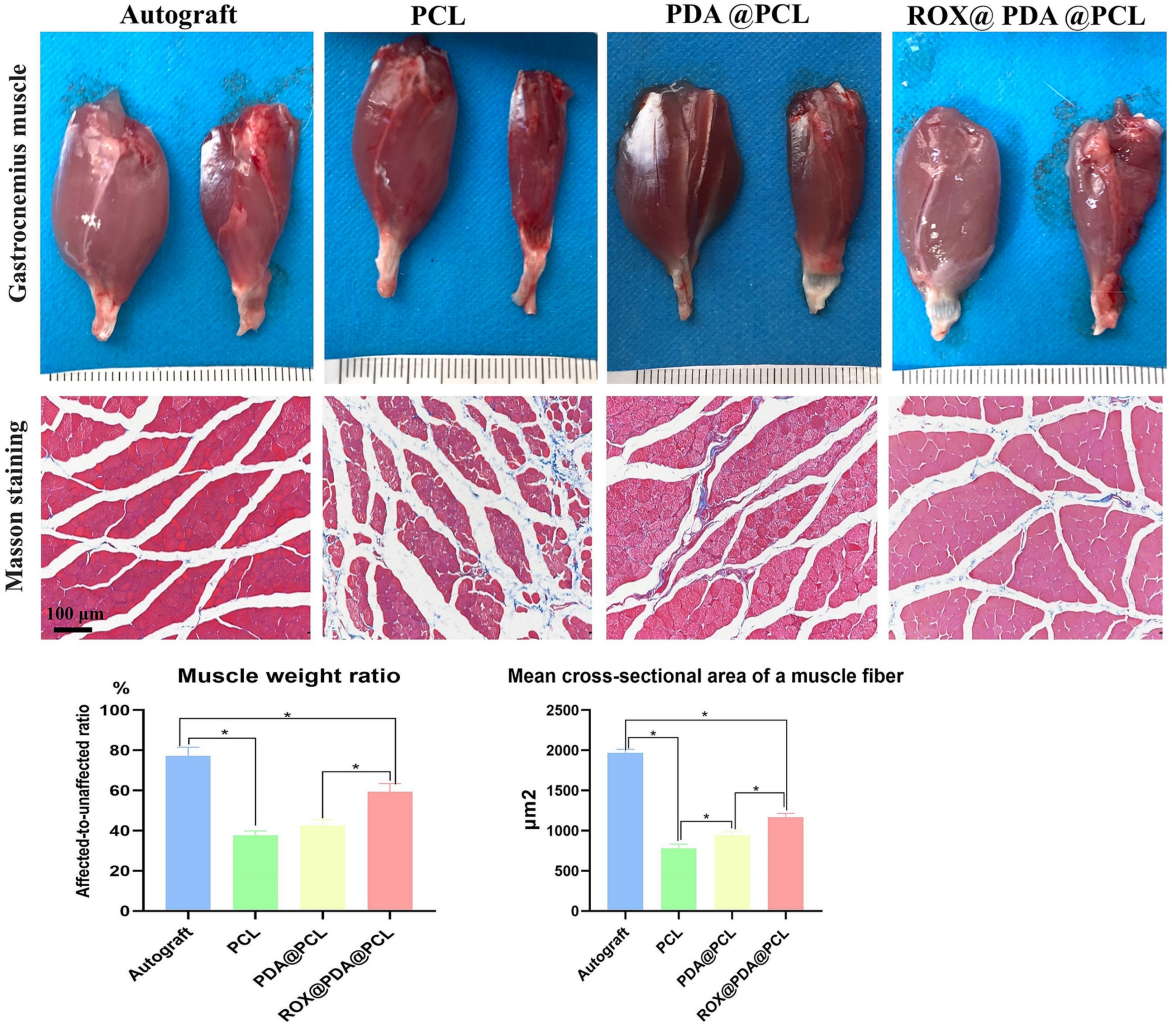
Muscle recovery. Compared with the contralateral side, gastrocnemius of all groups showed different degree of atrophy after nerve injury repair, but the atrophy degree and cross-sectional area of the ROX@PDA@PCL group were significantly lower than that of group PDA@PCL and PCL. In the gross view images of the gastrocnemius muscle, the left side of each image pair represents the normal gastrocnemius muscle, while the right side corresponds to the surgical-side gastrocnemius muscle. Data are expressed as mean ± SD, *n* = 6. *p < 0.05.

The mean cross-sectional area of a muscle fiber in the Autograft (*n* = 6), ROX@PDA@PCL (*n* = 6), PDA@PCL (*n* = 6), and PCL (*n* = 6) groups were 1967.75 ± 42.43 μm^2^, 1166.75 ± 49.57 μm^2^, 940.75 ± 45.37 μm^2^, and 779.25 ± 52.32 μm^2^, respectively. The cross-sectional area of the ROX@PDA@PCL group was significantly higher than the other conduit groups (*p* < 0.05), but significantly lower than that of the Autograft group (*p* < 0.05). Compared to the PDA@PCL and PCL groups, the muscle fibers in the ROX@PDA@PCL group were more tightly arranged, with less collagen fiber filling between muscle clusters (Figure 8).

## Discussion

In this study, a vascularized ROX@PDA@PCL nerve conduit was prepared for repairing sciatic nerve defects in rats. Through behavioral, electrical and histological tests, it was found that it could significantly improve the recovery of nerve function, and its repair effect of nerve defects was significantly better than that of groups PCL and PDA@PCL, but it still failed to achieve the repair effect of autologous nerve transplantation.

Nerve defects represent a category of disorders associated with a high disability rate (16, 17). The incidence of peripheral nerve defects, stemming from traffic accidents, earthquakes, iatrogenic injuries, and other factors, has been escalating annually. Notably, a significant proportion of trauma-induced peripheral nerve injuries affect young adults. Without effective intervention, these patients may suffer from perpetual motor dysfunction, sensory abnormalities or loss, and may even develop painful neuromas (18, 19). Such consequences impose immense psychological and economic burdens on both society and families.

The repair of peripheral nerve injuries constitutes a multifaceted pathological process, aiming to reconstruct the sensory and motor functions of the affected organs and mitigate the adverse impact of nerve defects on patients’ quality of life. The findings of this study underscore that autologous transplantation remains the gold standard for treating neurological deficits (20). However, this approach entails the loss of donor nerve function and is limited by the scarcity of available donor nerves (21).

Conversely, the ROX@PDA@PCL vascularized nerve conduit developed in this study emerges as a promising alternative. It effectively addresses nerve defects, with nerve conduction function and target organ function recovery approaching that observed in autologous transplantation. This innovative nerve conduit stands as an exemplary advancement in the field. In this study, we have designed a sophisticated guided drug-loaded nerve conduit. The intricate tissues of the biological body often possess directional growth structures, enabling them to optimize their biological functions to their fullest potential. Consider, for instance, the musculoskeletal system, which relies on the precise, directional parallel arrangement of muscle cells to orchestrate muscle contraction and relaxation with remarkable precision (22). Similarly, the vascular system leverages its directional architecture to efficiently transport blood to various target organs, facilitating material exchange vital for life processes. Analogously, the peripheral nervous system operates on its directional anatomical structure to swiftly transmit nerve impulses, ensuring seamless communication between the body’s various parts.

Despite advancements in understanding tissue structures, a considerable disparity remains between the efficacy of scaffolds that merely mimic the directional anatomical structure of tissues and the outcomes achieved through autologous nerve repair. To bridge this gap, we have incorporated ROX, a potent pro-angiogenic drug, into a PCL nerve conduit to construct a biodegradable vascularized nerve conduit. This enhancement significantly improves the nerve regeneration capabilities of the basic guiding conduit (PDA@PCL, or PCL conduit).

The conduit’s potential degradation mechanism involves PDA breaking down under the influence of pH and glutathione, triggering the release of ROX *in vivo* (23, 24). Subsequently, the exposed PCL undergoes hydrolysis and enzymatic degradation, ultimately leading to the complete biodegradation of the scaffold (25). Our findings reveal that the ROX@PDA@PCL group exhibited notably higher CMAP amplitudes and nerve conduction velocities compared to the PDA@PCL and PCL groups. The nerve conduction velocity is intricately linked to the thickness of the newborn nerve myelin sheath, while the CMAP amplitude correlates with the number of muscle fibers at the neuromuscular junction. A higher amplitude signifies a greater number of muscle fibers effectively connected to the nerve, and a faster conduction velocity indicates a thicker, more robustly regenerated nerve myelin sheath, leading to superior functionality. Consequently, the amplitude of CMAP and the nerve conduction velocity serve as indirect yet reliable indicators of neurological function recovery.

After sciatic nerve injury in rats, atrophy inevitably sets in, stemming from the deprivation of innervation to the affected muscles (26, 27). This leads to a profound impact on the rats, manifesting as limb paralysis, toe atrophy, and deformation. The examination of rat footprints serves as a telling indicator (28) of nerve re-innervation and functional recovery, offering a window into their therapeutic progress.

Remarkably, the sciatic nerve function index of the ROX@PDA@PCL group significantly outperformed that of other non-autologous transplantation groups. This suggests that the vascularized bionic nerve conduit employed in this group effectively rejuvenates the motor function of the lower limbs. Furthermore, the ROX@PDA@PCL group exhibited less collagen filling within muscle fibers and a larger cross-sectional area of these fibers, indicative of the new nerves exerting a more pronounced nutritional effect on the muscles.

The ROX@PDA@PCL conduit’s promotional effect on nerve regeneration may be attributed to its ability to strengthen the interplay between blood vessels and nerves. The synergistic relationship between nerve regeneration and angiogenesis has been extensively validated in the nervous system (29–33). In the Wallerian degeneration process following nerve injury, blood vessel formation precedes the migration of Schwann cells. This sequence of events is supported by various studies. Hobson et al. (34) found that Schwann cells in well-vascularized areas of rats exhibited superior migration and axonal regeneration rates compared to those in poorly vascularized areas. Cattin et al. (7) discovered that, following sciatic nerve transection, macrophages—under hypoxic stimulation—promoted angiogenesis by secreting VEGF-A. These new blood vessels induced Schwann cells to migrate along them, ultimately guiding the regeneration of axons.

Studies have shown that activating angiogenesis can significantly improve the recovery of nerve function (35–37). Huang et al. (38) fabricated a dual-layer conduit containing VEGF-A-transfected Schwann cells, which promoted peripheral nerve regeneration via angiogenesis in rats. Pan et al. (39) transplanted exogenous macrophages to a segmental nerve injury to enhance nerve repair by inducing angiogenesis.

The present study, while groundbreaking in its exploration, is not without limitations. Specifically, the intricate mechanism through which the ROX@PDA@PCL conduit fosters angiogenesis remains uncharted, warranting further investigation into its operational mechanisms. Nevertheless, our efforts have yielded meaningful results. We have demonstrated the restoration of motor function, neuroelectrophysiology, neurohistology, and muscle histology in the affected limbs of rats 12 weeks post-operation. This comprehensive analysis has clearly elucidated the therapeutic potential of the ROX@PDA@PCL vascularized bionic conduit in addressing sciatic nerve defects.

A limitation of this study is that the mechanism by which the ROX@PDA@PCL conduit promotes angiogenesis has not been explored in depth. However, we have demonstrated the restoration of motor function, neuroelectrophysiology, neurohistology, and muscle histology in the affected limbs of rats 12 weeks after the operation, thereby clarifying the therapeutic effect of the ROX@PDA@PCL vascularized bionic conduit on sciatic nerve defects. We will conduct a more detailed study to further investigate the biological mechanism by which the ROX@PDA@PCL vascularized biomimetic conduit promotes nerve regeneration in future work.

## Conclusion

The ROX@PDA@PCL vascularized bionic conduit has the potential to significantly advance peripheral nerve regeneration. It exhibits unique characteristics in promoting vascular regeneration, which may offer a novel therapeutic strategy for treating peripheral nerve defect.

## Data Availability

The original contributions presented in the study are included in the article/supplementary material, further inquiries can be directed to the corresponding authors.
